# Prenatal diagnosis of Pallister-Killian syndrome using cord blood samples

**DOI:** 10.1186/s13039-019-0449-x

**Published:** 2019-08-30

**Authors:** Ting Wang, Congmian Ren, Dan Chen, Jian Lu, Li Guo, Laiping Zheng, Yuan Liu, Hanbiao Chen

**Affiliations:** 1 Medical Genetic Center, Guangdong Women and Children Hospital, 521 Xingnan Avenue, Panyu, Guangzhou, China; 2 Ultrasound Diagnosis Department, Guangdong Women and Children Hospital, 521 Xingnan Avenue, Panyu, Guangzhou, China

**Keywords:** Pallister-Killian syndrome, Prenatal diagnosis, Isochromosome 12p, Cord blood, Ultrasound findings

## Abstract

**Background:**

Pallister-Killian syndrome (PKS) (OMIM:#601803) is a rare sporadic genetic disorder characterized by multi-malformations which is caused by the presence of the extra isochromosome 12p. PKS is featured by the tissue-limited mosaicism of the isochromosome 12p [i(12p)]. There were a wide spectrum of prenatal ultrasound findings of PKS, which made it difficult to be found in first or second trimester. Polyhydramnios, diaphragmatic hernia, and rhizomelic limb shortening were the most common prenatal ultrasound abnormalities in PKS. This study retrospectively analyzed the ultrasound findings and molecular cytogenetic results of four PKS fetuses diagnosed by using cord blood samples.

**Results:**

The ultrasound anomalies of four PKS fetuses are described as follows: fetal macrosomia, cerebral ventriculomegaly, increased NT thickness, rhizomelic limbs shortening, polyhydramnios. Biparietal diameter (BPD), head circumference (HC), abdominal circumference (AC), femur length (FL) measurements were above the mean in three fetuses,while one fetus showed rhizomelic limbs shortening. Combined with this study and previous literature, polyhydramnios was the most frequent anomaly observed in prenatal ultrasound examination of PKS, which accounted for 48% (94/194). Fetal macrosomia was present in 15% (29/194), cerebral ventriculomegaly in 13% (25/194), thickened nuchal fold in 9% (18/194), rhizomelic limbs shortening in 26% (51/194). I(12p) was found in the karyotype analysis of cultured cord blood lymphocytes and the mosaic ratios ranged from 2 to 5%. Single nucleotide polymorphisms array (SNP-array) results suggested that the whole short arm of chromosome 12 was duplicated with 2~3 copies. Fluorescence in situ hybridization (FISH) was performed to confirm the results of karyotype and SNP-array.

**Conclusions:**

In case non-specific indicators such as fetal macrosomia, polyhydramnios and rhizomelic limbs shortening are observed meanwhile in prenatal ultrasound, targeted detection of PKS should be considered. In the prenatal diagnosis of PKS, the combination of SNP-array and FISH with conventional karyotype are the key to seek i(12p) and for precise diagnosis.

## Background

Pallister-Killian syndrome (PKS) (OMIM:#601803) is a rare sporadic genetic disorder characterized by developmental delays, prominent foreheads, sparse frontotemporal hairs, craniofacial deformities, mental retardation, epilepsy, low tension, skin pigmentation, congenital heart defects and other systemic abnormalities. The feature of this syndrome is the presence of isochromosome 12p [i(12p)] mosaicism with tissue-limited. Tissue-limited mosaicism refers to the differences in chromosome between different tissues in the same individual. The percentage of cells with i(12p) depends on the tissue examined, regardless of the severity of the syndrome [1]. The mosaicism rates of skin fibroblasts, amniotic cells or chorionic villus cells are higher than that of rapidly dividing lymphocytes [2]. Over time, tetraploid cells are rapidly replaced by euploidy cells in the peripheral blood, and postnatal infants were usually diagnosed by buccal smear or skin fibroblasts.

Prenatal diagnosis of PKS remains a challenge because of the difficulty in detecting additional i(12p) and the diversity of mosaicism level. Moreover, the supernumerary marker chromosomes are easily decreased in amniotic fluid cell culture [3]. Using traditional cytogenetic banding techniques to identify marker chromosomes may be difficult. The most commonly considered prenatal diagnosis usually occurred in cases of congenital diaphragmatic hernia (CDH) or incidental chorionic villus sampling (CVS) or amniocentesis due to advanced maternal age, nonspecific ultrasound abnormalities or even less in the case of prenatal screening abnormalities [4]. There were a wide spectrum of prenatal ultrasound findings in PKS, which made it difficult to be found in first or second trimester. Polyhydramnios, CDH, and rhizomelic limb shortening were the most common prenatal ultrasound abnormalities in PKS [5].

Single nucleotide polymorphisms array (SNP-array) can detect copy number variations using DNA isolated from uncultured specimens [6]. In this study, we presented 4 cases of PKS diagnosed by karyotype and SNP-array in second trimester. All the 4 cases were analysed in terms of ultrasound manifestations carefully and compared with the previous literature. To our knowledge, this series is the first report of prenatal diagnosis of PKS in China from a single center.

## Methods

We retrospectively analyzed the ultrasound and genetic results of all fetuses diagnosed with PKS in our medical genetic center between January 2017 and December 2018. The follow-up were performed by experienced nurse. We reviewed the ultrasound findings of PKS published in the last decade in order to identify the anomalies and markers existing in PKS. This study obtained the informed consent of all involved women and was approved by the Ethics Committee of Guangdong Women and Children hospital.

Voluson E8 (GE Healthcare, USA) was applied for ultrasound examinations, which were performed by specialists experienced in fetal medicine. The gestational weeks of all the involved women exceeded 26 weeks when they were referred to our center. Subsequent cordocentesis were performed and 2 mL cord blood were aspirated into asepsis pipe and sent to the laboratory as soon as possible. All specimens were divided into two parts, one for cell culture and one for molecular genetic testing after DNA isolation using QIAamp DNA Blood Mini Kit (QIAGEN, Germany). NANODROP 2000 (Thermo, USA) was applied to determine DNA concentration. CytoScan 750 K chip (Affymatrix, USA) was used for SNP-array. All the procedures were operated according to the manufacturer’s protocol. The results were analyzed with ChAS3.1.0.15 (Affymatrix, USA) software. The remaining samples were employed for routine cell culturing according to standard culture process. Karyotypes were obtained after GTG-banding. Fluorescence in situ hybridization (FISH) was performed to confirm the diagnosis of PKS using chromosome 12p telomere probe (Abbott Vysis TelVysion 12p Spectrum Green probe, USA) and control chromosome 16 centromere probe (Abbott Vysis CEP 16 Spectrum Orange probe, USA).

## Results

**Fig. 1:**
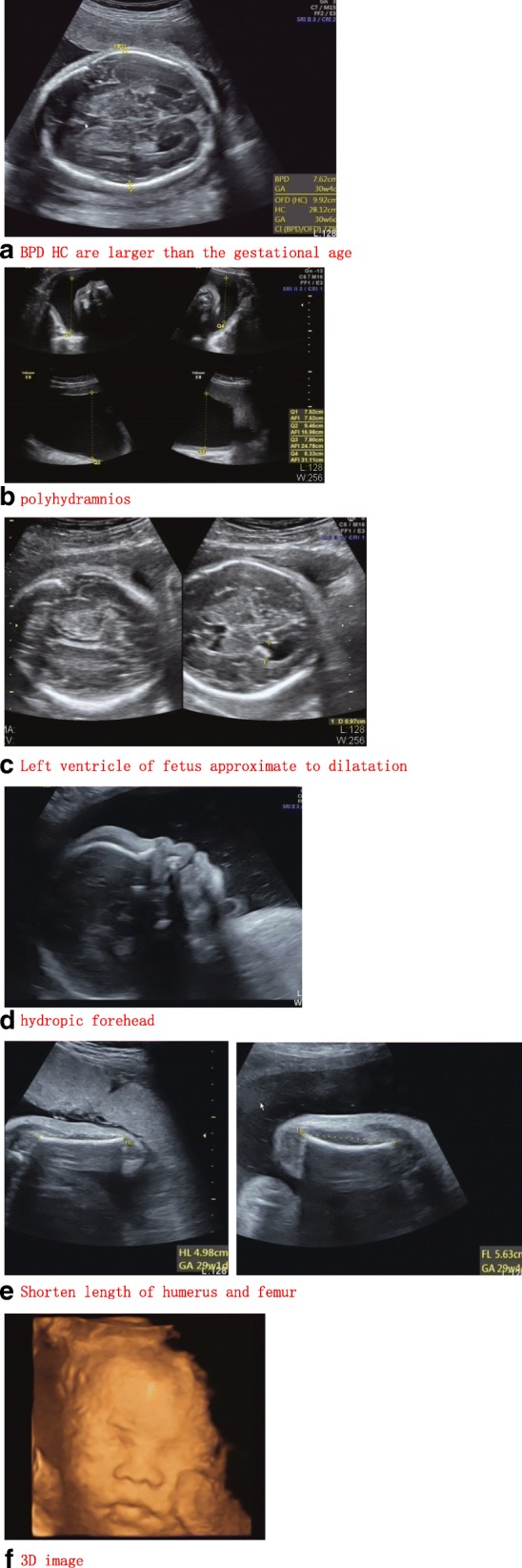
Ultrasound image of 4 fetuses with PKS. **a**: 2 of 4 fetuses’ biparietal diameter and head circumference were large for gestational age and (**b**) polyhydramnios; **c**: Besides mild polyhydramnios, fetus2’s left ventricle approximate to dilatation. **d**: Fetus4 has hydropic forehead and (**e**) shorten length of humerus and femur. **f**: There are no sinificant positive characteristics in all the fetuses’profiles of 3D ultrasound images

**Fig. 2:**
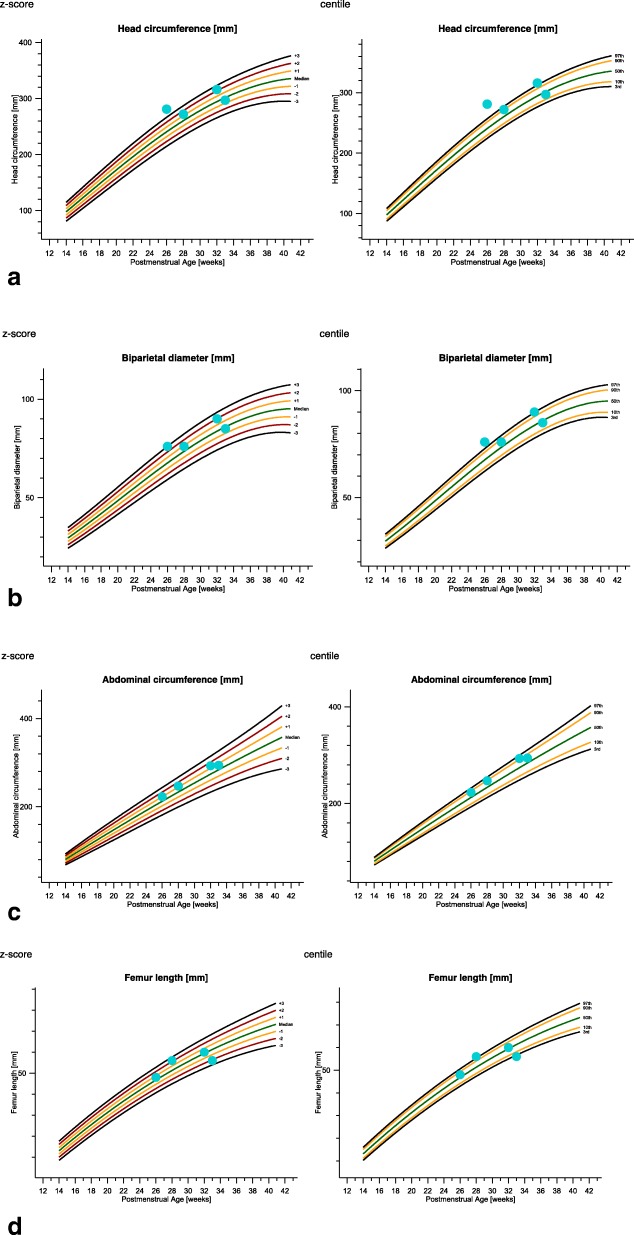
Fetal biometry charts of fetal growth based on INTERGROWTH-21st International Standards for Fetal Growth (v1.6.4). Biparietal diameter, head circumference, abdominal circumference and femur length measurements were above the mean in three fetuses, while fetus4 showed rhizomelic limbs shortening. **a**: Z-scores and centile of head circumference. **b**: Z-scores and centile of biparietal diameter. **c**: Z-scores and centile of abdominal circumference. **d**: Z-scores and centile of femur length

The mean age of the women was 32 years and the mean gestational age was 30 weeks. All the pregnancies were singleton and the women had unremarkable history. Two women were primigravida and the other had healthy children. Anomalies of ultrasound findings were as followed: fetal macrosomia, cerebral ventriculomegaly, increased NT thickness, rhizomelic limbs shortening, polyhydramnios. The descriptions of ultrasound findings were summarized and demonstrated in Table 1 and Fig. 1. In this study, the most common ultrasound finding was polyhydramnios that affected all 4 fetuses which was consistent with previous literature [3]. Table 2 and Fig. 2 documented the Z-scores and Centile of 4 fetuses with PKS according to International Standards for Fetal Growth (v1.6.4). Biparietal diameter (BPD), head circumference (HC), abdominal circumference (AC) and femur length (FL) measurements were above the mean in three fetuses, while one fetus showed rhizomelic limbs shortening. Combined with our dataset and previous literature [3], polyhydramnios was the most frequent anomaly observed in ultrasound examination which accounted for 48% (94/194). Fetal macrosomia was present in 15% (29/194), cerebral ventriculomegaly in 13% (25/194), thickened nuchal fold in 9% (18/194), rhizomelic limbs shortening in 26% (51/194).

**Table 1:** Ultrasound findings of PKS fetus

	MOTHER AGE	GA(weeks)	BPD(mm)	HC(mm)	AC(mm)	FL(mm)	AFI(mm)	ultrasound anomaly
fetus1	40	26	76	281	223	48	357	fetal macrosomia, polyhydramnios
fetus2	35	28	76	272	247	56	311	cerebral ventriculomegaly, polyhydramnios
fetus3	20	32	90	316	293	60	392	fetal macrosomia, polyhydramnios
fetus4	33	33	85	297	294	56	311	polyhydramnios, thickened nuchal fold, rhizomelic limbs shortening

*GA* gestational age, *BPD* biparietal diameter, *HC* head circumference, *AC* abdominal circumference, *FL* femur length, *AFI* amniotic fluid index

**Table. 2:** Z-scores and centile of 4 fetuses with PKS

		Z-scores				centile			
	GA	HC	BPD	AC	FL	HC	BPD	AC	FL
fetus1	26	4.6	3	1	0.6	100	100	85	73
fetus2	28	1.3	0.9	1.2	2.2	90	80	88	99
fetus3	32	2.2	2	1.4	0.2	99	97	91	59
fetus4	33	−0.5	−0.3	0.7	−2.1	32	39	76	2

*GA* gestational age, *BPD* biparietal diameter, *HC* head circumference, *AC* abdominal circumference, *FL* femur length

**Fig. 3:**
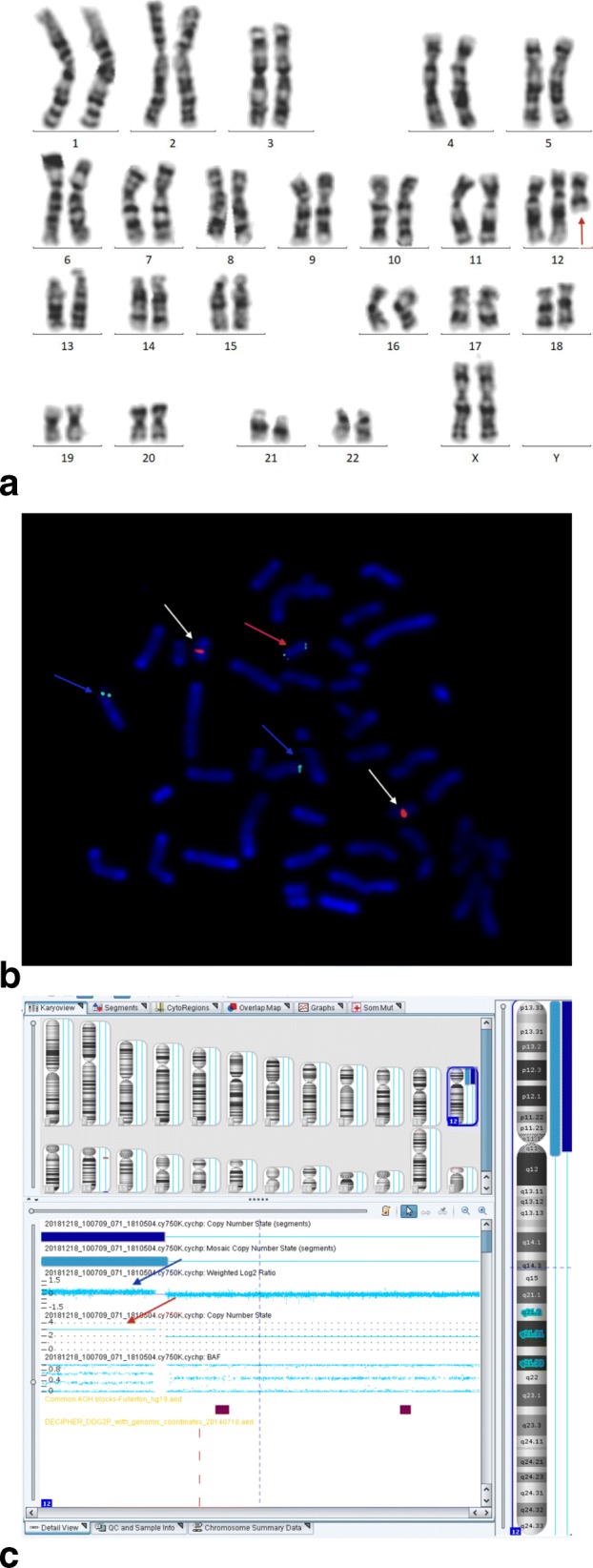
**a**: GTG-banding karyotype of PKS fetus in cord blood lymphocytes. Red arrow shows a supernumerary isochromosome 12p. **b**: Metaphase FISH using 12p telomere probe labeled green and 16 centromere probe labeled orange. Blue arrows show the normal chromosome 12. White arrows show the chromosome 16. Red arrow shows the isochromosome 12p. **c**: SNP-array analysis of uncultured cord blood. Blue arrow shows the log2 ratio. Red arrow shows the lines of copy number variants of 12p were between 2 and 3

Since the gestational weeks were all over 24 weeks, all 4 cases received invasive prenatal diagnosis with cordocentesis. Karyotype, SNP-array and FISH results of 4 fetuses were shown in Fig. 3 and summarized in Table 3 according to the International System for Human Cytogenomic Nomenclature 2016 version (ISCN 2016). Karyotype analysis of cultured cord blood lymphocytes was performed in all cases, and the cells counting was increased to 100 cells as a diagnostic test. De novo supernumerary i(12p) was found in the cultured cord blood lymphocytes and the mosaic ratios ranged from 2 to 5%. DNA extracted from uncultured cord blood lymphocytes was used for SNP-array detection and the results suggested that the whole short arm of chromosome 12 was duplicated with a copy number between 2 and 3. Interphase FISH was performed to confirm the results of karyotype and SNP-array. The mosaic ratios of i(12p) in uncultured cord blood interphase FISH ranged from 22 to 46%. After genetic counseling, all women decided to terminate their pregnancies.

**Table. 3:** Molecular and cytogenetic results of 4 fetuses with PKS

	karyotype	SNP-array	FISH
fetus1	mos 47, XY, +i (12) (p10) [5]/46, XY [95]	arr [GRCh38] 12p13. 33p11. 1 (173786_34835641) × 2–3	nuc ish 12p13 (TEL × 4)/12p13 (TEL × 2) [23/77]
fetus2	mos 47, XX, +i (12) (p10) [3]/46, XX [97]	arr [GRCh38] 12p13. 33p11. 1 (173786_34835641) × 2–3	nuc ish 12p13 (TEL × 4)/12p13 (TEL × 2) [18/82]
fetus3	mos 47, XX, +i (12) (p10) [5]/46, XX [95]	arr [GRCh38] 12p13. 33p11. 1 (173786_34835641) × 2–3	nuc ish 12p13 (TEL × 4)/12p13 (TEL × 2) [35/65]
fetus4	mos 47, XX, +i (12) (p10) [2]/46, XX [98]	arr [GRCh38] 12p13.33p11. 1 (173786_34835641) × 2–3	nuc ish 12p13 (TEL × 4)/12p13 (TEL × 2) [22/78]

## Discussion

PKS is difficult to detect in prenatal diagnosis because most cases were sporadic. Less than 100 prenatal cases have been published since first described by Gilgenkrantz in a prenatal diagnosis of tetrasomy 12p from amniocytes [7]. Santamaria et al. published a prenatal case report of a fetus with massive diaphragmatic hernia and summarized the main ultrasound indicators of PKS including polyhydramnios, rhizomelic limbs shortening and CDH [8]. Kucińska-Chahwan et al. believed that the presence of CDH is sufficient to modify routine to targeted testing for PKS [9]. Mourali et al. presented the first case of early prenatal diagnosis of PKS due to the presence of increased nuchal translucency and CDH [10]. However, none of the 4 cases we presented had CDH. This deviation likely reflects the diversity of clinical expression of PKS. Doray et al. stated that the most frequent indicator of PKS was polyhydramnios (84%) [11]. Polyhydramnios seen along with CDH may be the result of esophageal compression due to hernia [12]. On the contrary, all the fetuses we presented had polyhydramnios but not accompanied with CDH. This indicated that polyhydramnios in PKS may be caused by multi-malformation.

Fetuses with PKS exhibited a very typical growth pattern characterized by increased BPD and HC, usually above the 90th percentile, accompanied with femoral growth delay, which is significantly below the 10th percentile for gestational age. In our study, the HC percentile of four fetuses was 100th, 90th, 99th, and 32th respectively. But the percentile of FL was higher than 10th, except that of fetus 4 (2th). This further illustrated the varity of ultrasound manifestations of PKS. Karaman et al. presented 15 children with PKS, two of whom were macrosomia [13]. Salzano et al. summarized all the previously published reports of PKS and obtained the percentage of fetal macrosomia was 14% (26/190) [3]. The ultrasound findings of fetus 1 and fetus 3 were decribed as fetal macrosomia shown in Table 2 due to the high percentile of HC and AC. Compared with previous literatures, 2 cases of macrosomia were found in 4 cases of PKS fetus in this study, with higher proportion. Although the number of cases was relatively small, it also showed the difficulty of PKS in prenatal diagnosis. In case of the wide phenotypic spectrum of PKS, we strongly recommend that even if each of these abnormal ultrasound findings is non-specific, PKS should be highly suspected when rhizomelic limbs shortening, polyhydramnios and fetal macrosomia are observed simultaneously. Thicken nuchal fold and cerebral ventriculomegaly were also found in our study, which were consistent with the ultrasound findings reported in previous literature [14, 15].

PKS is caused by the presence of additional i(12p). It is difficult to detect i(12p) cells in cord blood due to the low response of lymphocyte in phytohemagglutinin (PHA) stimulation [16, 17]. I(12p) were usually absence in lymphocytes culture, but could be found in skin fibroblasts and other tissues such as buccal smears, chorionic villi and amniotic cells [18–20]. Four pregnant women had missed the time of amniocentesis when they were refered to our center, so they could only take cordocentesis for prenatal diagnosis. Theisen et al. used genomic DNA to detect i(12p) that could not be detected in karyotype analysis of PHA-stimulated blood culture by aCGH [21]. Unlike to previous published literatures, we also performed cytogenetic and molecular analysis of all the four fetuses with PKS using cord blood samples. Since SNP-array results indicated the presence of 12p with 2 to 3 copies, the cell counts of karyotype were expanded to 100 and the tetrasomy cells of 12p were finally found as Fig. 3 shown. FISH on uncultured cord blood cells confirmed the presence of 12p mosaicism in four fetuses.The results of SNP-array in cord blood of 4 fetuses showed 2 to 3 copies of 12p, which may be due to the neutralization of double dose of 12p in normal cells and quadruple dose of 12p in abnormal cells. In the prenatal diagnosis of PKS, the combination of SNP-array and FISH with conventional karyotype are the key to seek i(12p) and for precise diagnosis.

The mechanism of i(12p) formation is still not fully revealed. Previous literatures have illustrated that maternal meiotic II nondisjunction is a mechanism of tetrasomy 12 mosaicism, although there were several reports of paternal nondisjunction [22, 23]. Similar to other autosomal aneuploidy syndrome, advanced maternal age is a high risk factor. Theoretically, in case a parent had i(12p) germline mosaicism or balanced rearrangement on chromosome 12p, there were high risk of recurrence of pregnancy with PKS [24]. The four couples also had no chromosome rearrangements such as cryptical pericentric inversions which could lead to duplication or isochromosome formation of 12p [25].

In conclusion, our study presented the atypical expression of PKS in prenatal diagnosis such as ultrasound finding and diagnostic samples. In case non-specific indicators such as fetal macrosomia, polyhydramnios and rhizomelic limbs shortening are observed meanwhile in ultrasound examination, targeted detection of Pallister-Killian syndrome should be considered, including expanded cell counts in karyotype and using uncultured genomic DNA for molecular detection.

## Data Availability

The datasets used and/or analyzed during the current study are available from the corresponding author on reasonable request.

## Abbreviations

AC: Abdominal circumference
AFI: Amniotic fluid index
BPD: Biparietal diameter
CDH: Congenital diaphragmatic hernia
FISH: Fluorescence in situ hybridization
FL: Femur length
GTG-banding: Giemsa trypsin banding
HC: Head circumference
i(12p): Isochromosome 12p
PHA: Phytohemagglutinin
PKS: Pallister-Killian syndrome
SNP-array: Single nucleotide polymorphisms array
